# Expression of Hsp70, Igf1, and Three Oxidative Stress Biomarkers in Response to Handling and Salt Treatment at Different Water Temperatures in Yellow Perch, *Perca flavescens*

**DOI:** 10.3389/fphys.2017.00683

**Published:** 2017-09-12

**Authors:** Nour Eissa, Han-Ping Wang, Hong Yao, Zhi-Gang Shen, Adel A. Shaheen, Elsayed N. Abou-ElGheit

**Affiliations:** ^1^Aquaculture Genetics and Breeding Laboratory, Ohio State University Piketon, OH, United States; ^2^Department of Immunology, College of Medicine, University of Manitoba Winnipeg, MB, Canada; ^3^Department of Fish Diseases and Management, Faculty of Veterinary Medicine, Benha University Toukh, Egypt; ^4^Aquatic Diseases Laboratory, Aquaculture Division, National Institute of Oceanography and Fisheries Cairo, Egypt

**Keywords:** stress responses, aquaculture practice, sub-lethal effects, metabolic enzymes, fish, gene expression, salinity, husbandry stressors

## Abstract

Stress is a major factor that causes diseases and mortality in the aquaculture industry. The goal was to analyze the expression of stress-related biomarkers in response to different stressors in yellow perch, which is an important aquaculture candidate in North America and highly sensitive to handling in captivity. Three fish groups were established, each having four replicates, and subjected to water temperatures of 14, 20, and 26°C and acute handling stress was performed followed by a salt treatment for 144h at a salinity of 5 ppt. Serum and hepatic mRNA levels of heat shock protein (hsp70), insulin-like growth factor 1 (Igf1), glutathione peroxidase (Gpx), superoxide dismutase 1 (Sod1), and glutathione reductase (Gsr) were quantified at seven times interval over 144 h using ELISA and RT-qPCR. Handling stress caused a significant down-regulation in Hsp70, Gpx, Sod1, and Gsr at a water temperature of 20°C compared to 14 and 26°C. Igf1 was significantly upregulated at 20°C and down-regulated at 14 and 26°C. Salt treatment had a transient reverse effect on the targeted biomarkers in all groups at 72 h, then caused an upregulation after 144 h, compared to the control groups. The data showed a negative strong regulatory linear relationship between *igf1* with *hsp70* and anti-oxidative gene expressions. These findings could provide valuable new insights into the stress responses that affect fish health and could be used to monitor the stress.

## Introduction

The study of the candidate genes of the stress responses could be unique signatures or imprints of specific stressors and determine early signs of stressors. Aquaculture and fisheries industries have several unavoidable stressors, such as handling, transportation, temperature, crowding, salinity, hyperoxia, and hypoxia that result in the stress responses of fish (Eissa and Wang, 2016). Stress responses of fish to variable stressors include a highly ordered set of responses regulated by the neural, endocrine, and immune systems. The primary function of these responses is to compensate fish biological systems, arrange the metabolism to afford the energy required by the fish and maintain homeostasis (Tort, 2010; Tort and Teles, 2012). A broad range of metabolic processes and pathways are involved in this stress response (Wendelaar Bonga, 1997; Barton, 2002; Tort and Teles, 2012), which refer to that some stress-related genes in several organs and tissues, such as heart, liver, and muscles are expressed and rapidly changed, followed by the synthesis of proteins involved in the adaptation process (Staib et al., 2007).

Traditionally, stress biomarkers in fish include plasma cortisol, glucose, hematological and hydro-mineral measurements (Barton, 2002; Tort, 2010; Eissa and Wang, 2013; Eissa et al., 2013). However, in some circumstances, there are difficulties interpreting results due to the stress response being controlled by intrinsic and extrinsic factors, which may alter the results as negative or positive feedback mechanisms in the hormonal pathways (Pottinger, 2008). Although homeostatic adjustments of plasma and whole body stress hormones and metabolites in response to various stressors have been well studied, the underlying transcriptional regulation and functional genomics of stress responses can provide a better understanding of stress pathophysiology (Prunet et al., 2008; Aluru and Vijayan, 2009; Eissa and Wang, 2016). In our recent study on yellow perch stress response, the stress protocol under different water temperatures showed a fluctuant elevation in plasma cortisol concentration (Eissa and Wang, 2013). Therefore, identifying stress biomarkers using genomic tools is an essential aspect for minimizing fish disease and mortality in the aquaculture industry and fisheries stock enhancement.

Heat shock proteins (HSPs) comprise a group of highly conserved proteins that have general protective function and play a vital role in cellular homeostasis in all living organisms (Yamashita et al., 2010; Tkáčová and Angelovičová, 2012). Animals express HSPs in response to stressful stimuli (Parsell and Lindquist, 1993; Hofmann, 1999). Under stress, HSPs act as molecular chaperones to regulate protein homeostasis, to prevent aggregation and to assist in refolding of misfolded proteins (Rye et al., 1999; Ranford et al., 2000). High expression of *hsp70* mRNA has been observed in fish subjected to overcrowding (Gornati et al., 2004, 2005), transport stress (Poltronieri et al., 2007), and heat stress (Bertotto et al., 2011). Many authors stated that gene expression of stress proteins is modulated in response to stress, and transcriptional responses of these genes can be used as sensitive biomarkers in bio monitoring of aquatic environments (Rungrassamee et al., 2010; Zhou et al., 2010; Sinha et al., 2012).

Reactive oxygen species (ROS) are formed naturally during oxidative metabolism (Roch, 1999). Stress can cause over-production of ROS and increase lipid peroxidation (LPO), which may affect cell viability through cell membrane destruction and enzyme deactivation (Nordberg and Arner, 2001; Circu and Aw, 2010). Then, cell apoptosis and oxidation of DNA and proteins may be enhanced, which may cause a variety of physiological disarrays, such as reduced disease resistance, immunosuppression, reduced growth and productivity (Pandey et al., 2003). The alteration of the oxidative system balance, if not adequately renovated by the antioxidant barrier or antioxidant system, prompts an oxidative stress with cellular damage, which makes the organism sensitive to pathological conditions (Lykkesfeldt and Svendsen, 2007). Glutathione peroxidase (*gpx*), superoxide dismutase (*sod1*), and glutathione reductase (*gsr*) are well-developed regulatory mechanisms protecting against oxidative stress. They can maintain the fish hemostasis by eliminating ROS when exposed to various stressors, especially temperature changes and salinity (Hansen et al., 2006; Mohankumar and Ramasamy, 2006; Zhang et al., 2010; Shaheen et al., 2014b). Therefore, oxidative stress biomarkers could be used in environmental monitoring programs. This area has been paid lots of attention (McCarthy and Shugart, 1990).

Long term and repeated stress are reported to have severe adverse effects on fish growth (Wendelaar Bonga, 1997; Bertotto et al., 2011). Growth is a biological phenomenon controlled by a complex of genes as insulin-like growth factors (IGFs) that show action in controlling fish growth (Gornati et al., 2004, 2005; Bertotto et al., 2011). Insulin-like growth factor 1 (*igf1*), is an important protein in the regulation of most physiological processes in fish, such as somatic growth and metabolism and is down-regulated by starvation and nutritional stress, and activates the IGF 1 receptor (*igf1r*) (Moriyama et al., 1994; Björnsson, 1997; Solberg et al., 2012). *igf1* genes have been characterized in several fish species, and the expression of its mRNA has been observed in tissues from larvae, fry and adults (Berishvili et al., 2006; Patruno et al., 2006, 2008; Sinha et al., 2012). The levels of growth-related factors in fish have often been discussed with nutritional and osmotic changes (Moriyama et al., 2000; Wilkinson et al., 2006; Reinecke, 2010; Beckman, 2011). However, less attention has been given to the effect of stressors, such as husbandry or environmental stressors, on the expression levels of growth related genes in fish (Deane and Woo, 2009; Reinecke, 2010). It is imperative to illuminate the influence of stress at the molecular level of fish growth to improve fish production and health under captivity and culture conditions. The regulation and transcriptional expression of these stress and growth related genes are prized indicators of physiological states in aquatic species (Shaheen et al., 2014a). They provide the potential to comprehend the mechanisms, which respond to various stressors, and provide the possibility for assessing stress responses in different conditions and for a further search for the functionally related genes (Dahlhoff, 2004; Krasnov et al., 2005; Eissa and Wang, 2016).

Handling, temperature changes, and salinity are common stressors in the aquaculture industry and is one of most common husbandry stressors (Eissa and Wang, 2013, 2016). The handling stress disrupts the fish homeostasis through the alteration of metabolic pathways that are involved in various biological processes including the immune responses against invaders (Gonzalez et al., 2007; Wiseman et al., 2007). Therefore, appropriate immune responses play a vital role in the adaptive mechanism supporting cellular homeostasis post-stressor exposure (Eissa and Wang, 2016). Although, some fish species can tolerate large a wide range of temperature changes (Cossins et al., 2006). Other fish species can tolerate the variation in their water temperature and are more susceptible to stress, which has detrimental effects on fish health (Dominguez et al., 2004; Gollock et al., 2006). Furthermore, salinity is a critical contributor to the stress in fish and alleviates negative effects on the fish health and homeostasis (An et al., 2010). Although, yellow perch is susceptible to a wide range of stressors (Jentoft et al., 2002; Defo et al., 2014; Grasset et al., 2014; Fadhlaoui and Couture, 2016). Handling, temperature changes, and salinity are common stressors in the aquaculture practices of yellow perch (Eissa and Wang, 2013).

Yellow perch (*Perca flavescens*) is a very important aquaculture and recreational fish species in North America. This species is highly sensitive to handling and disturbances in captivity and intensive culture conditions (Head and Malison, 2000). Handling mortality is often high and has been one of the factors that limit Yellow Perch aquaculture and stock enhancement. Moreover, previously we reported that handling, temperature and salt treatment altered the cortisol responses in yellow perch (Eissa and Wang, 2013). In this study, we hypothesized that handling stress and water temperature or/and salt modulate the stress-associated genes in yellow perch. To test our hypothesis, protein and mRNA expression levels of *hsp70, igf1, gpx3, sod1, and gsr* in serum and liver tissues of yellow Perch subjected to stressors of handling and salt treatment at different water temperatures were quantified. Information on the biomarkers associated with various stressors in this species could provide valuable new insights into the stress responses that affect fish survival in the aquaculture and recreational fisheries industry.

## Materials and methods

This study and all experimental procedures involving animals were performed according to the protocol approved by the Ohio State University Institutional Animal Care and Use Committee.

### Experimental fish

Nine hundred sixty Yellow Perch (48 ± 10 g) were obtained from the Aquaculture Research Center, Ohio State University South Centers, Ohio, the USA. Fish were held at water temperatures of 8–10°C in 800-L experimental tanks and fed twice daily to satiation with a commercial diet before transfer. Two weeks before experimentation, fish were transferred to twelve 400 L experimental tanks (80 fish/tank) to acclimate to the experimental system and target water temperatures of 14, 20, and 26°C. Fish were acclimated by increasing the temperatures gradually until reaching the target temperature for each group, and then the final temperature was maintained.

### Experimental design

#### Handling at different temperatures

**Figure 1:**
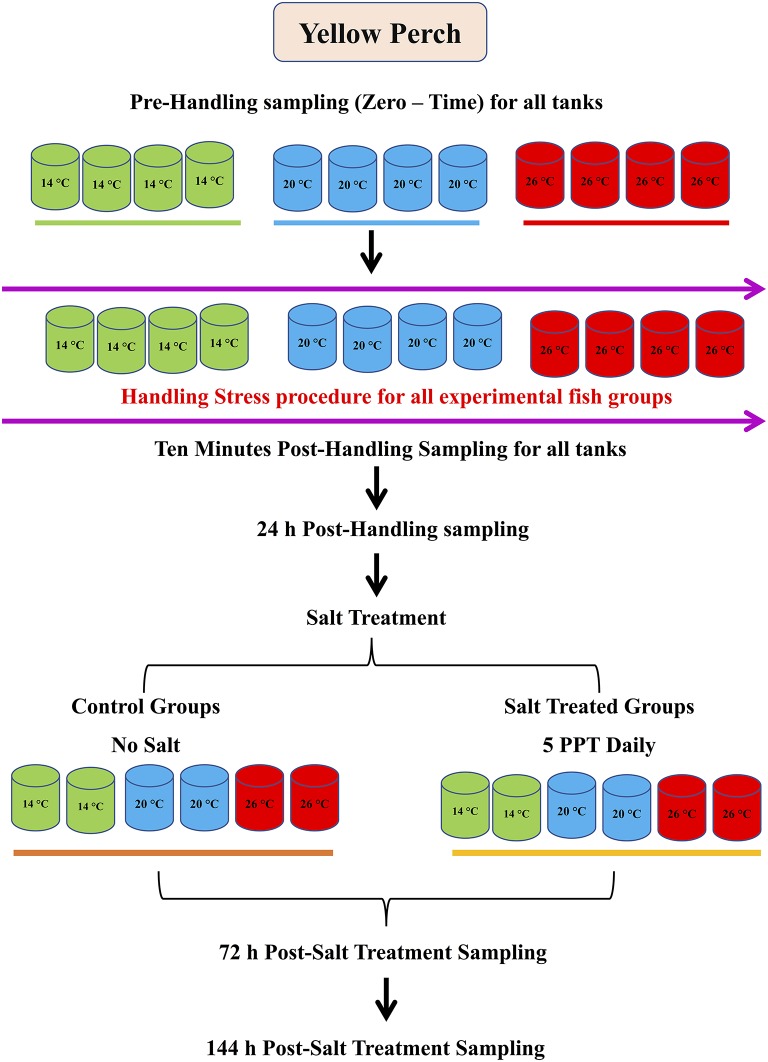
Experimental design and sampling points.

There were three experimental groups (four replicates each, 80 fish/replicate), which were subjected to three water temperatures: 14, 20, and 26°C (Figure 1). Water temperatures were adjusted through increasing it gradually (1°C/day) until reaching the target temperature for each group and the final water temperatures were maintained throughout the experiment. Stress handlings were conducted for all groups tank by tank, by weighing all fish using standard practices. Before weighing fish, half of the water from the tank was siphoned into an empty tank for holding weighed fish. After weighing, fish were returned to the original tank by netting, and then the water was returned to the tank. The same manner and time were applied to all tanks. Handling time was monitored and recorded for each tank. This procedure was carried out at 10–11 a.m., and the timing for one feeding was adjusted 1 h after handling. During this study, fish were sampled before the handling (zero time, pre-handling samples), immediately after handling (10 min after the stress handling was finished), and 24 h post-handling (Figure 1).

#### Salt treatment

In aquaculture practice, aquaculturists usually treat fish with salt (5%) after handling to eliminate bacteria and disease. Therefore, to mimic this practice, 1 h after samples were collected for the handling stress, the fish in two tanks of each experimental group (other two tanks as control) were subjected to daily salt treatment (ST) at a salinity of 5 ppt for 6 days. The salt treatment procedure was performed daily by adding 5 ppt to treat fish in tanks for 2 h: weighed salt for each tank was dissolved in a large bucket with the same water temperature; water flow in each tank was stopped right before the salt water was added; half of the salt water was added to each tank at the beginning, then another half an hour later. Water flow returned at the end of 2 h at the rate of 1 L/Min. Fish were sampled at 72 and 144 h after the last salt treatment.

#### Blood and liver samples

Three fish per replicate (nine fish per treatment) were carefully netted and were euthanized using tricaine methanesulfonate (MS222) at 250–350 mg/L in water for blood and tissue sampling. Blood samples were drawn near the caudal peduncle using 1-cc U-100 syringes without anticoagulant (Becton–Dickinson, Franklin Lakes, NJ, USA) and transferred into Eppendorf tubes and allowed to clot at room temperature in a slanting position. The blood samples were subsequently used to obtain serum (by centrifugation at 10,000 g for 5 min at 4°C) and stored in clean centrifuge tubes at −80°C until used for assaying. Fish were carefully dissected, and liver samples were taken and rinsed with phosphate-buffered saline (PBS 1%, pH 7.4) to remove any red blood cells and clots. The tissues were weighed and homogenized (10x the volume) in 50 mM PBS containing 1 Mm EDTA (pH 7.4). The homogenates were then centrifuged (20 min, 13,200 g, 4°C) and the supernatants were separated for protein assays.

#### Protein analyses

The protein levels of Hsp70, Igf1, Gpx, Sod1, and Gsr in serum and liver tissue homogenates were measured spectrophotometrically (BioTek's Epoch™, USA) using colorimetric kits (Cayman Chemical, USA) and (MyBioSource, Inc. San Diego, CA, USA) according to the manufacture instructions. The total protein concentrations in liver homogenates were quantified by Bradford protein assay (BioRad, CA, USA). Subsequently, the protein levels of Hsp70, Igf1, Gpx, Sod1, and Gsr were calculated based on the protein concentration in the sample.

#### RNA extraction and reverse transcription

Liver samples were collected from yellow perch and immediately immersed in RNA-Later® (Ambion®, Life Technologies, USA). Samples were stored at −80°C until analysis. For RNA extraction, tissue samples were removed from RNA-Later and homogenized by tissue homogenizer and extracted using TRIZOL® reagent according to the manufacturer's instructions (Ambion®, Life Technologies, USA). The extracted RNA was re-suspended in 50 μL of DNAse/RNAse-free water and quantified using a Nanodrop spectrophotometer (Nd-1000, NanoDrop Technologies, DE, USA) at 230, 260, 280, and 320 nm to obtain estimates of RNA quantity and quality. Total RNA was stored at −80°C in single use aliquots. One microgram of RNA from each sample were treated with RQ1 RNase-Free DNase® (Promega® Corporation, USA) according to the manufacturer's instructions and then reverse transcription was carried out using a High Capacity cDNA Reverse Transcription Kit with RNase Inhibitor (Invitrogen™, USA) according to the manufacturer's instructions in a Bio-Rad® Thermo cycler at 25°C for 10 min, followed by 37°C for 120 min, and 85°C for 5 min, then cooled to 4°C. cDNA samples were stored at −20°C for RT-qPCR.

#### Quantitative real-time PCR (RT-qPCR)

Expression of stress-related genes that include heat shock protein 70kDa (*hsp70*) and insulin like growth factor 1 (*Igf1*) as well as three oxidative stress genes, glutathione peroxidase 3 (*gpx3*), glutathione reductase (*gsr*), and superoxide dismutase 1 (*sod1*) were assessed through quantitative polymerase chain reaction (qPCR). The sequence of primers was used as published previously (Table 1) (Martin et al., 2017). Primers were tested and validated using NCBI BLAST and Integrated DNA Technologies' Oligoanalyzer 3.1. RT-qPCR reactions were performed in triplicate using SYBR® Select Master Mix (Applied Biosystems®, USA) on a 7,500 Real-Time PCR (Applied Biosystems®, USA) according to the manufacturer's instructions. cDNA and primers were added to SYBR® Select Master Mix with a final volume of 20 μl. Melt curve analysis was performed to ensure the amplification of the single product. β-actin was selected as the endogenous normalizer for subsequent analyses, for each of the target genes tested. The corresponding pre-handling sampling point (before handling directly) for each temperature was used as a control sample for the corresponding experimental groups. The relative gene expressions were calculated using 2^−ΔΔCt^ method (Livak and Schmittgen, 2001). Fold change percent for each gene was normalized against the pre-handling sampling point.

**Table 1:** Real-time quantitative polymerase chain reaction and assay conditions for genes in *Perca flavescens*.

**Target gene**	**Primer**	**Sequence (5^′^–3^′^)**	**PCR product length (base pairs)**	**Accession number**
*gpx3*	Sense	TGACTACACGGGCAAGAGTG	128	FJ826525.1
	Antisense	GGAAGCCAAGAAGGGTGAG		
*igf1*	Sense	CGCAGGGCACAAAGTGGAC	102	AY332492.2
	Antisense	CCC AGT GTT GCC TCG ACTTG		
*gsr*	Sense	CTGGTGTGGATGTGTGGAAG	98	HQ206482.1
	Antisense	CGAACTTCTCCTCGTCGTTC		
*sod1*	Sense	GCATGTAGGAGACTTGGGCAAT	64	KT783483.1
	Antisense	CCGTGATTTCTATCTTGGCAACA		
*hsp70*	Sense	TGTTGGTCGGTGGCTCAA	60	KX050165.1
	Antisense	TTGAAGAAGTCCTGAAGCAGCTT		
*β-actin*	Sense	GCCTCTCTGTCCACCTTCCA	62	AY332493.2
	Antisense	GGGCCGGACTCATCGTACT		

#### Statistical analysis

Two-Way analysis of variance (ANOVA) followed by multiple comparison tests was used for testing mean differences between groups at the different time points at significance levels (*P* ≤ 0.05). Since the data were not passed the Shapiro-Wilk normality test, Spearman's test was carried out to determine the correlation between the mRNA expression levels of liver for all genes. GraphPad Prism version 7 was used for all statistical analysis and creating the graphs.

## Results

### Handling by temperature interactions on Hsp70 and Igf1

**Figure 2:**
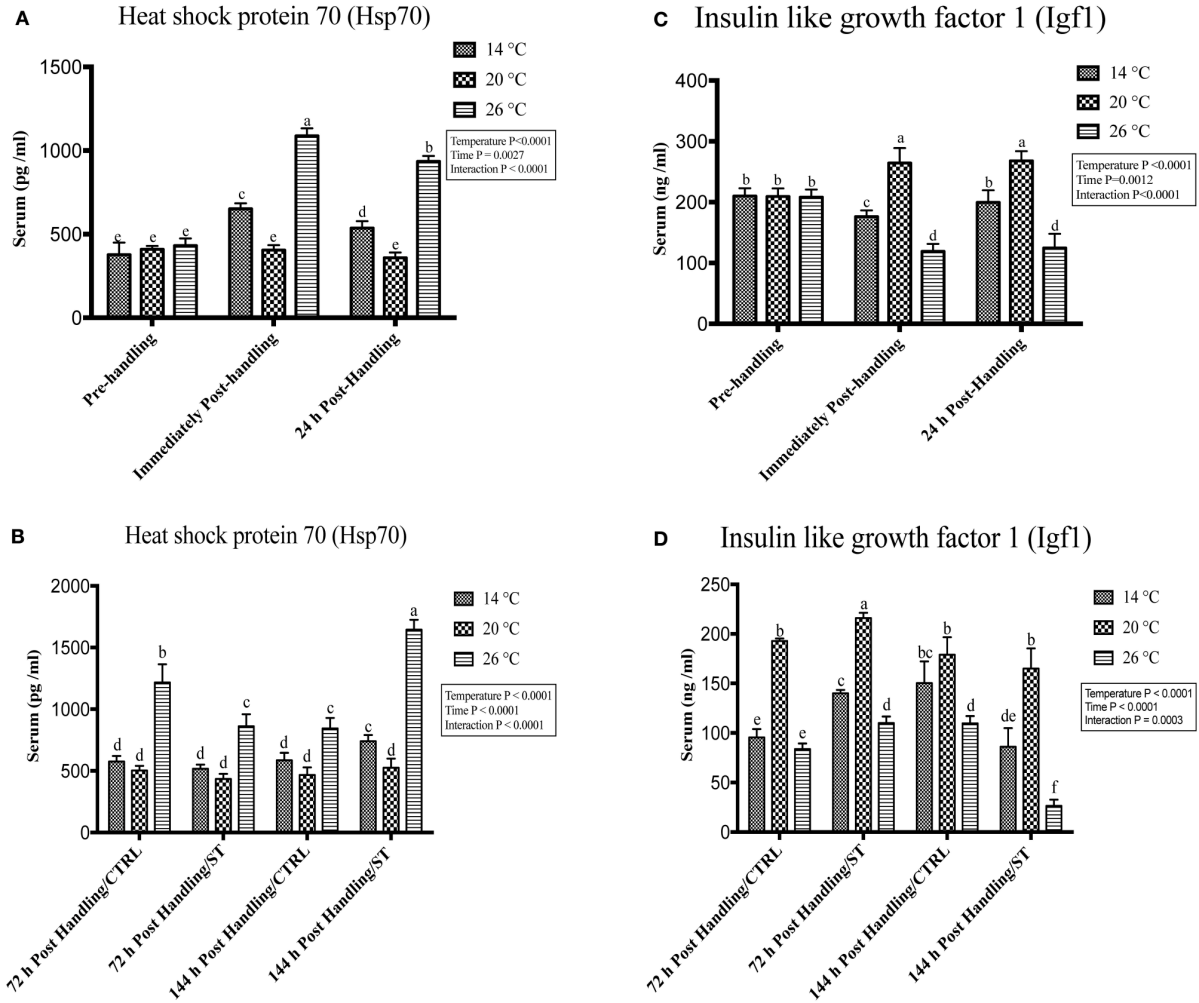
Effects of handling and salt treatment at different water temperatures of yellow perch, *Perca flavescens* on serum Hsp70 and Igf1 protein levels. Serum protein levels of **(A,B)** Heat Shock Protein (Hsp)-70 and **(C,D)** insulin like growth factor 1 (Igf1). Hsp70 and Igf1 were quantified using ELISA. Two-way ANOVA analysis followed by multiple comparison tests was applied. Different letters denote a significant difference. *n* = 9–12/group. Data represented Mean ± SD.

**Figure 3:**
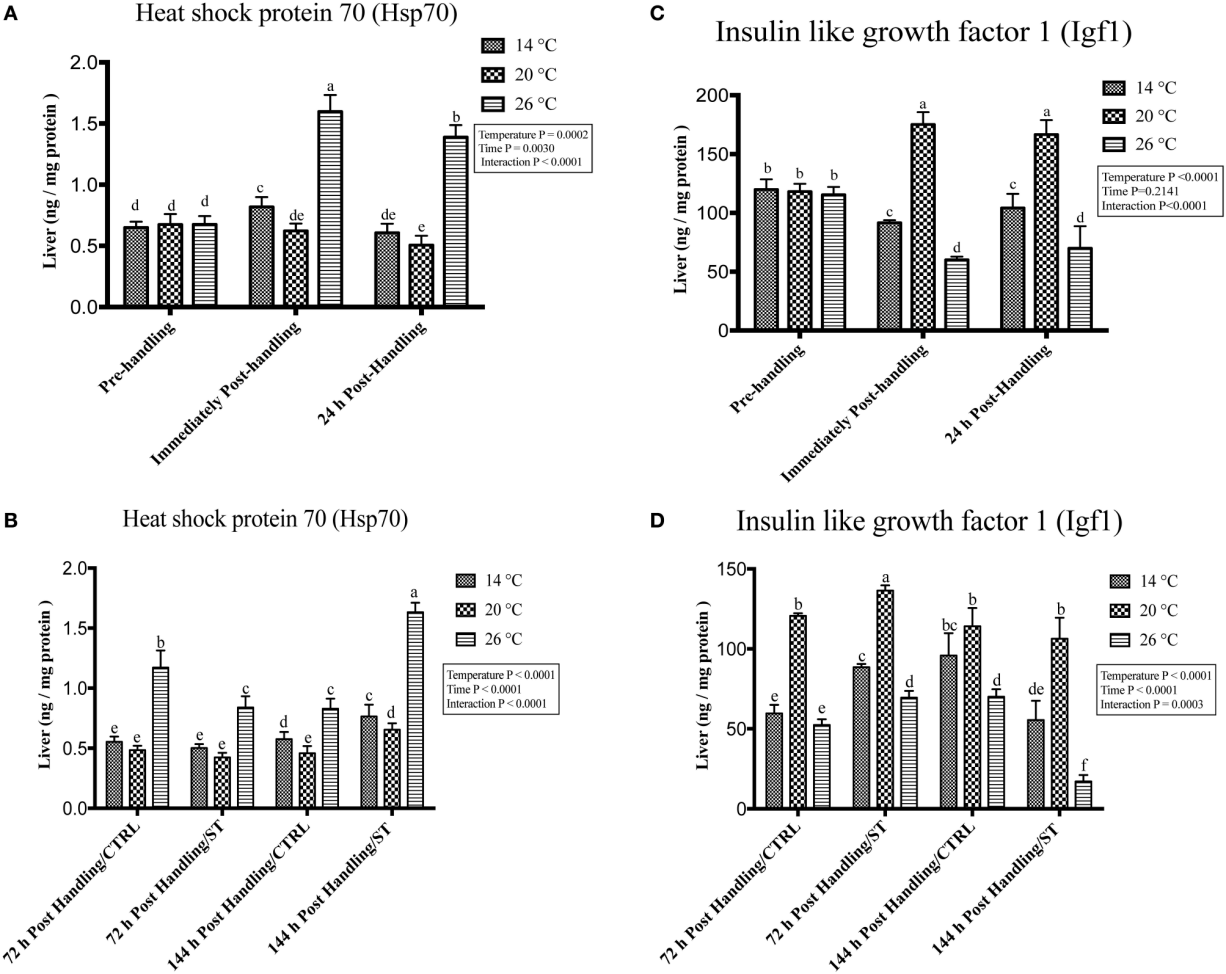
Effects of handling and salt treatment at different water temperatures of yellow perch, *Perca flavescens* hepatic Hsp70 and Igf1 protein levels. sProtein levels of **(A,B)** Heat Shock Protein (Hsp)-70 and **(C,D)** insulin like growth factor 1 (Igf1) in liver homogenates. Hsp70 and Igf1 were quantified using ELISA. The total protein concentrations in liver homogenates were quantified by Bradford protein assay. Two-way ANOVA analysis followed by multiple comparison tests was applied. Different letters denote a significant difference. *n* = 9–12/group. Data represented Mean ± SD.

**Figure 4:**
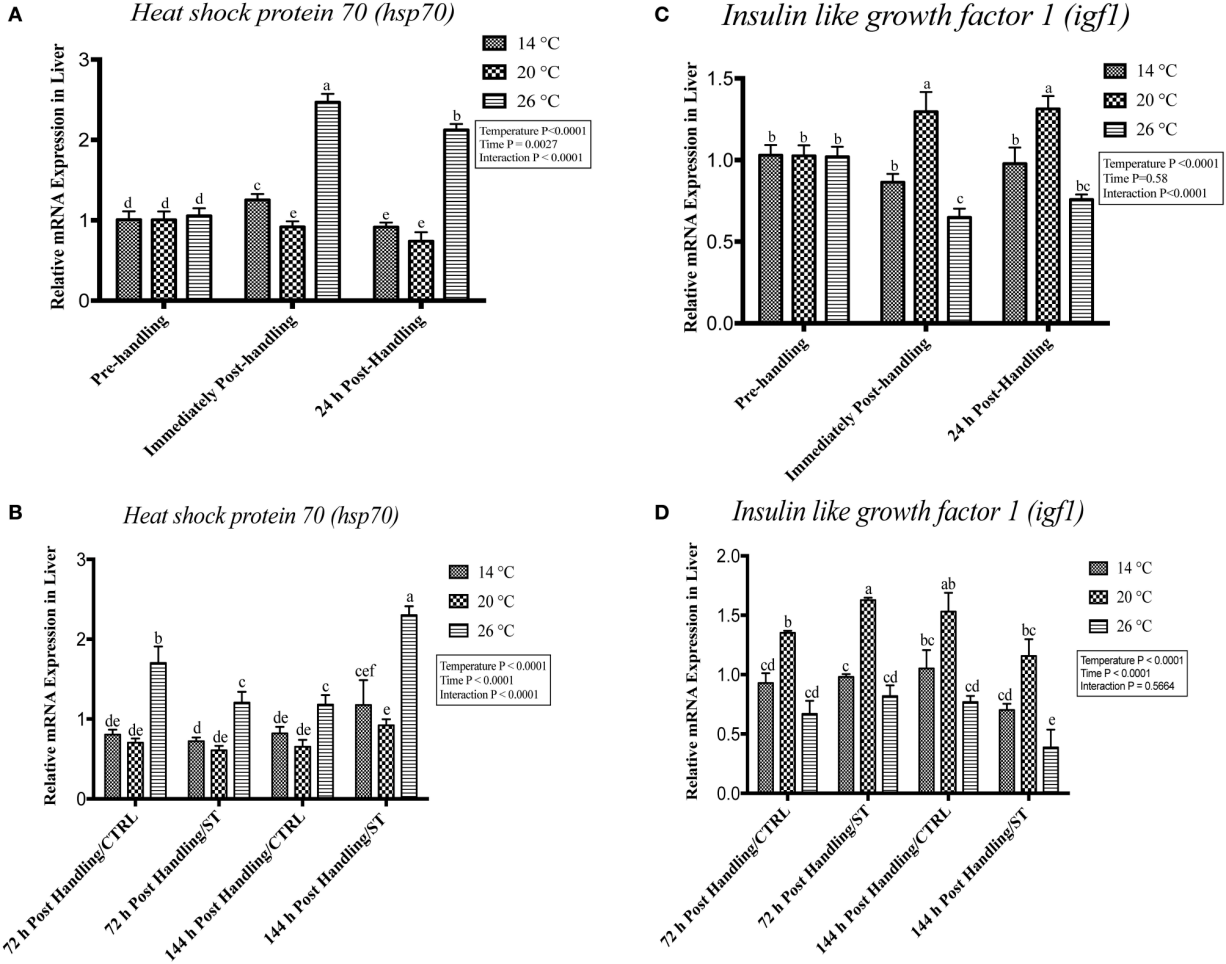
Effects of handling and salt treatment at different water temperatures of yellow perch, *Perca flavescens on* hepatic *hsp70 and igf1* mRNA levels. mRNA levels of **(A,B)** Heat Shock Protein (*hsp*)-70 and **(C,D)** insulin like growth factor 1 (*igf1*) in the liver. *hsp70 and igf1* were quantified using RT-qPCR. Two-way ANOVA analysis followed by multiple comparison tests was applied. Different letters denote a significant difference. *n* = 9–12/group. Data represented Mean ± SD.

Exposure to handling stress at different water temperatures caused significant (*P* ≤ 0.05) effects at three tested water temperatures at time dependent manner. Immediately post-handling, serum, and hepatic protein level of Hsp70 and hepatic mRNA level of *hsp70* up-regulated at water temperature 14 and 26°C while significantly down-regulated at 20°C (Figures 2A, 3A, 4A). Twenty-four hours after handling, Hsp70 expression declined in all groups compared to the immediate post handling time point (Figures 2A, 3A, 4A).

Handling stress at different temperatures negatively impacted the protein and mRNA expression levels of Igf1; immediately after handling, interestingly, serum and hepatic levels of Igf1 increased at 20°C while decreased significantly at 14 and 26°C (Figures 2C, 3C, 4C). The expression level exhibited 24 h post-handling was significantly increased than immediately after handling at water temperature 14 and 26°C with no difference was reported at water temperature 20°C (Figures 2C, 3C, 4C).

Moreover, the analysis showed that the serum and hepatic expression level of Hsp70 and Igf1 were affected significantly by the temperature, time and their interactions (*P* < 0.05) (Figures 2A,C, 3A, 4A). Surprisingly, the time effect did not show significance at hepatic protein and mRNA levels of Igf1 (Figures 3C, 4C).

### Handling by temperature interactions on expression of oxidative stress genes

**Figure 5:**
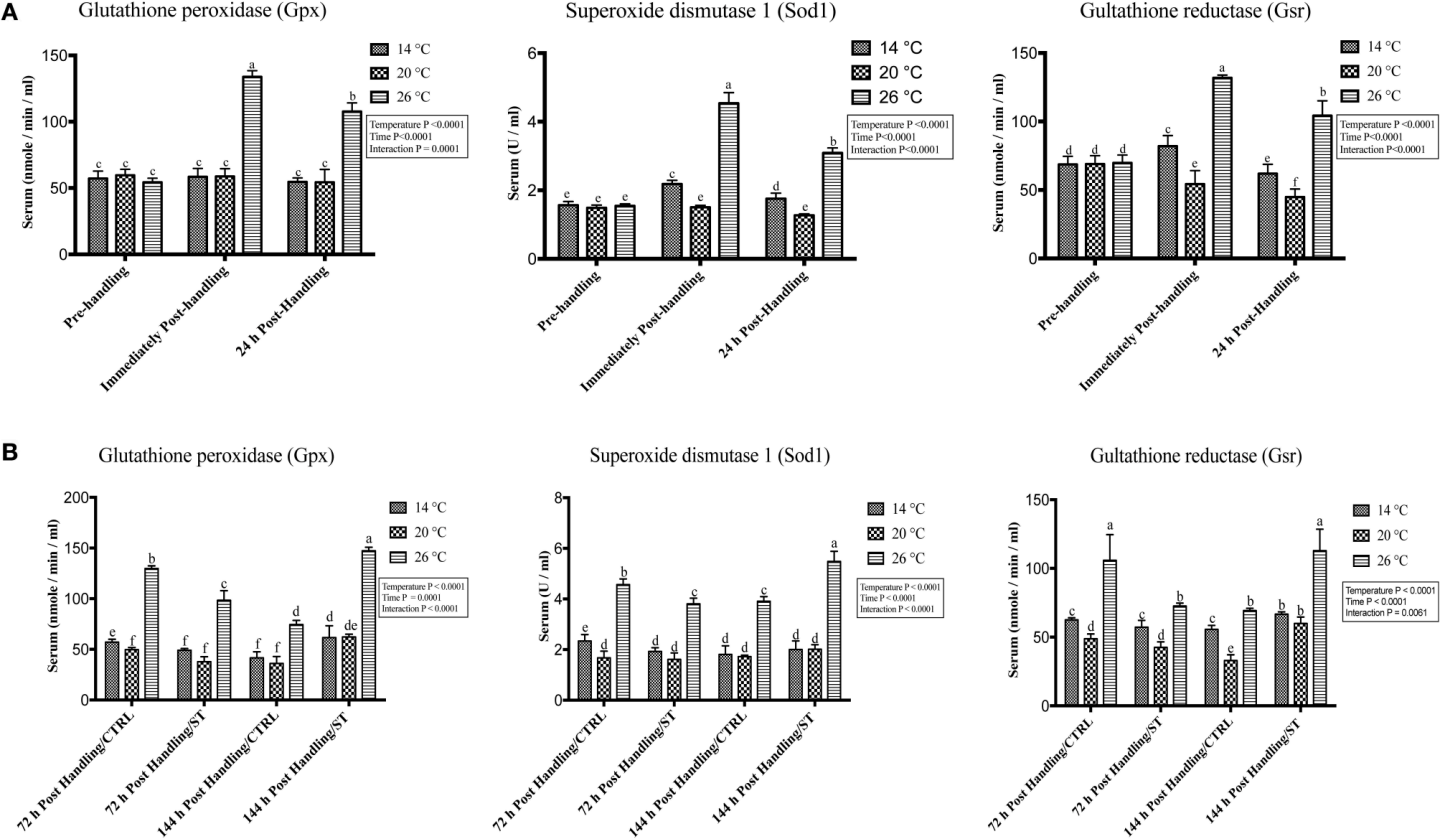
Effects of handling and salt treatment at different water temperatures of yellow perch, *Perca flavescens* on serum level of oxidative stress markers. Serum levels of oxidative stress markers **(A,B)**, glutathione peroxidase (Gpx), superoxide dismutase 1 (Sod1) and glutathione reductase (Gsr). Serum protein levels of Gpx, Sod1, and Gsr were quantified using ELISA. Two-way ANOVA analysis followed by multiple comparison tests was applied. Different letters denote a significant difference. *n* = 9–12/group. Data represented Mean ± SD.

**Figure 6:**
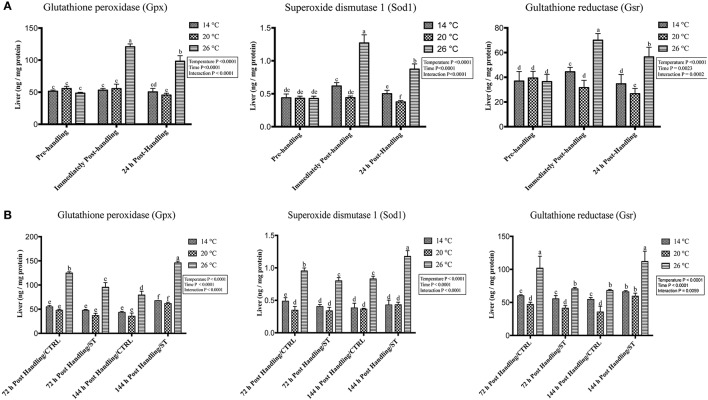
Effects of handling and salt treatment at different water temperatures of yellow perch, *Perca flavescens* on hepatic protein level of oxidative stress markers. Protein levels of oxidative stress markers **(A,B)**, glutathione peroxidase (Gpx), superoxide dismutase 1 (Sod1) and glutathione reductase (Gsr) in liver homogenates. Protein levels of Gpx, Sod1, and Gsr were quantified using ELISA. The total protein concentrations in liver homogenates were quantified by Bradford protein assay. Two-way ANOVA analysis followed by multiple comparison tests was applied. Different letters denote a significant difference. *n* = 9–12/group. Data represented Mean ± SD.

**Figure 7:**
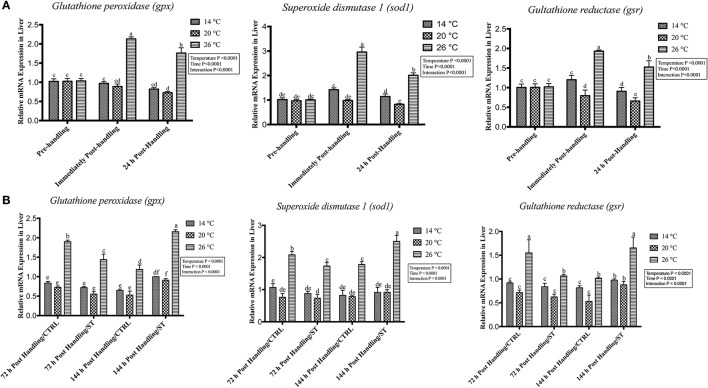
Effects of handling and salt treatment at different water temperatures of yellow perch, *Perca flavescens* on hepatic mRNA level of oxidative stress markers. mRNA levels of oxidative stress markers **(A,B)**, glutathione peroxidase 3 (*gpx3*), superoxide dismutase 1 (*sod1*) and glutathione reductase (*gsr*) in liver homogenates. mRNA levels were quantified using RT-qPCR. Two-way ANOVA analysis followed by multiple comparison tests was applied. Different letters denote a significant difference. *n* = 9–12/group. Data represented Mean ± SD.

The oxidative stress genes exhibited different levels of expression after handling at different temperatures. Immediately post-handling stress, serum and hepatic protein level of oxidative stress markers (Gpx, Sod1, Gsr) and hepatic mRNA levels (*gpx3, sod1, gsr*) showed a significant up-regulation at water temperature of 26°C compared to 14 and 20°C water temperatures while 24 h post-handling stress, their expression levels decreased at 26°C compared to post-handling time point with significant down-regulation at 20°C in (Figures 5A, 6A, 7A). The temperature effect, time effect, and their interactions showed a significance for the oxidative stress genes (*P* < 0.05) (Figures 5A, 6A, 7A).

### Effects of salt treatment on Hsp70 and Igf1

Salt treatment caused transient down-regulation of serum and hepatic protein levels of Hsp70 as well as hepatic mRNA levels (*hsp70*) after 72 h of treatment compared to the 72 h control groups especially at water temperature 26°C (Figures 2B, 3B, 4B). Hsp70 expression of salt treated group declined significantly at a group of water temperature 26°C while no significant decrease at 14 and 20°C water temperatures (Figures 2B, 3B, 4B) was detected. Continuation of salt treatment up to 144 h significantly impacted the serum and hepatic Hsp70 levels in all water temperatures and resulted in its upregulation; the highest up-regulation level was recorded at 26°C (Figures 2B, 3B, 4B).

The igf1 expression level in the serum and liver was affected by salt treatment. Salt treatment caused positive transient change through decrease the stress level, which represented in Igf1 at 72 h in treated group compared to the control one; but continuous salt treatment resulted in an increase of the stress level, which impacted the growth and was reflected in expression of Igf1 (Figures 2D, 3D, 4D). 72 h/ST groups exhibited upregulation of Igf1 at 20°C compared to 72 h/CTRL groups. At 20°C group, Igf1 was up regulated significantly compared to 14, and 26°C. Prolonged salt treatment resulted in down-regulation of *Igf1* in groups of all groups, especially at 26°C, where a significant down-regulatory impact on its expression in serum and liver (Figures 2D, 3D, 4D). Furthermore, the expression levels of Hsp70 and Igf1 during the salt treatment were significantly changed by the temperature, time and their interactions (*P* < 0.05) (Figures 2B,D, 3B,D, 4B,D).

### Effects of salt treatment on expression of oxidative stress genes

In this study, salt treatment had a transient stress mitigation effect within the first 72 h salt treatment only, while prolonged salt treatment for 144 h stimulated the oxidative stress marker expression that refers to increase the stress response level of yellow perch. The serum and hepatic protein level of oxidative stress markers (Gpx, Sod1, Gsr) and hepatic mRNA levels (*gpx3, sod1, gsr*) exhibited a clear expression pattern for salt treatment after handling at different temperatures (Figures 5B, 6B, 7B). All three markers had a lesser expression at 72 h/ST when compared with 72 h/Control, but after 144 h, the entire salt treated groups exhibited an increase in the expression level, indicating that salt became a stressful factor (Figures 5B, 6B, 7B). Gpx, Sod1, Gsr expression levels were significantly lower in 72 h/ST than 72 h/CTRL at 26°C, which reversed and increased after 144 h in the same fish group (Figures 5B, 6B, 7B). Oxidative stress markers showed no significant changes between water temperatures 14 and 20°C, while 72 h salt treatment resulted in transient lower *their expression* levels compared to other timing points, and their expression increased under continuous salt treatment (Figures 5B, 6B, 7B). Additionally, the temperature effect, time effect, and their interactions during the salt treatment showed a significance for the oxidative stress genes (*P* < 0.05) (Figures 5B, 6B, 7B).

### The relationship between Igf1 and stress related genes

**Figure 8:**
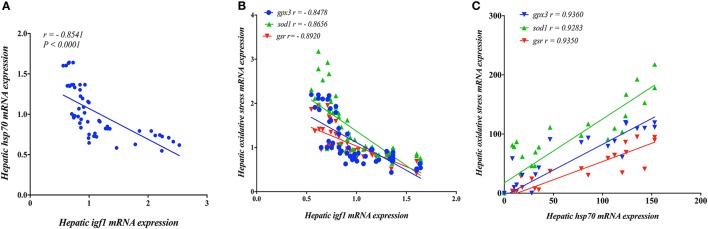
The relationship in the expression of Insulin Growth like Factor (*igf*)-1 with **(A)** Heat Shock Protein (*hsp70*) and **(B)** oxidative stress related genes (*gpx3, sod1, gsr*) in the liver of yellow perch. **(C)** The relationship between mRNA levels of hsp70 and oxidative stress related genes (*gpx3, sod1, gsr*) in the liver of yellow perch. Spearman's correlation was applied.

Next, we investigated the relationship between *igf1* and stress-associated genes. A strong significant negative linear regulatory relationship (*r* >−0.80) between *mRNA levels of igf1* as growth related gene and stress-associated genes (*hsp70, gpx3, sod1, gsr*), which refers to that high-stress level could reduce the growth rate, inverse relationship (Figures 8A,B). Moreover, a strong positive linear association (*r* > 0.90) between hsp70 and oxidative stress-associated genes (*hsp70, gpx3, sod1, gsr*) (Figure 8C).

## Discussion

In this study, the expression pattern of the potential stress-related biomarkers that associated with handling stress and salt treatment under different temperature in yellow perch were quantified. The present study revealed that physiological stress induced by handling stress and salt treatment at different temperatures can alter the expression of particular markers related to stress and growth in yellow perch and that a relationship may exist between Hsp70, Oxidative stress (GPx, SOD, and Gsr), and growth-related marker (Igf1) expression. Moreover, the water temperature is critical for yellow perch stress responses as the results showed that the handling stress and salt treatment magnified the stress-related biomarkers levels at the water temperature of 26°C, which is referring to this temperature unsuitable for yellow perch aquaculture. Salt treatment, a regular aquaculture practice, resulted in transient decreasing the stress level but its prolonged treatment could impact and increase the stress level. Therefore, this study provides new insights in the stress responses of yellow perch and highlights the potential use of hsp70, igf1 and oxidative stress-related genes as potential stress biomarkers.

The molecular chaperone Hsp70 is playing an important role in the stress process (Basu et al., 2002) and its upregulation as a result of one stressor might be able to afford a cross-protection of subsequent experiences of an additional stressor (Todgham et al., 2005). In the present study, handling stress and temperature impacted the serum and hepatic Hsp70 protein and hepatic mRNA levels significantly at the water temperature of 26°C while declined at 20 and 14°C. 24 h post-handling. There was a significant change in Hsp70 strength, as in all groups there was a lower expression of Hsp70 than immediately after handling. Temperature-induced increases in expression of Hsp70 in different species; in barramundi (*Lates calcarifer*) (Newton et al., 2012), the mRNA level of *hsp70* increased after crowding stress (Gornati et al., 2004; Caipang et al., 2008a; Salas-Leiton et al., 2010) have been reported. Changes in water temperaturesd (Place and Hofmann, 2005; Niu et al., 2008) lead to an upregulation of the expression of the inducible form of the heat shock protein 70 (*hsp70*) gene in *Dicentrarchus labrax* (European sea bass) when subjected to transport stress (Poltronieri et al., 2007). Therefore, up regulation of Hsp70 levels may serve as an early indicator of temperature stress in fish (Currie et al., 2000). Also, the time associated with fish water temperature exposure seems to be a key factor that should be given careful consideration. On the other hand, a study indicated that handling stress did not alter levels of hepatic Hsp70 in rainbow trout (*Oncorhynchus mykiss*) (Vijayan et al., 1997).

There is a physiological link between stress level and growth-related genes in the different fish species (Reinecke et al., 2005). Moreover, Igf1 is a major downstream gene responsive to growth hormone and involved in regulating growth (Tsai et al., 2014). Many studies have been shown a regulatory link between hormones and Hsp expression in different fish species and also Igf1 is critical in the regulation of hepatic HSP during stress adaptation (Deane et al., 1999; Ackerman et al., 2000; Sathiyaa et al., 2001). The current study reported a significant down-regulation of Igf1 at the water temperature 26 and 14°C, while a significant upregulation at 20°C after immediately after handling. Moreover, *igf1* correlated inversely with *hsp70*, as Hsp70 was down-regulated and Igf1 up-regulated at 20°C. These results agreed with many authors (Deane et al., 1999, 2000, 2002) confirming that there is a regulatory link between Hsp and Igf1. Other studies reported an increase of Igf1 under acute handling stress; in Atlantic salmon (*Salmo salar*) elevated Igf1 levels were observed after repeated acute stress (McCormick et al., 1998). Chinook salmon exhibited a higher degree of upregulation of Igf1 in the group maintained at a warm temperature than those maintained at an ambient temperature (Beckman et al., 1998). These studies suggest that the growth hormone (GH)– insulin like growth factor (Igf)-1 axis may provide a combined signal where favorable environments or unfavorable conditions for growth and development of fish would up-regulate or down-regulate this axis. Stressors have been reported to reduce the growth of fish, and also impact metabolism through the mechanism of glucocorticoid via the hypothalamus-pituitary-interrenal (HPI)-axis (Mommsen et al., 1999).

In this study, protein levels and genes associated with oxidative stress showed up-regulation when handling stress was applied at the water temperature of 26°C in both serum and liver of yellow perch, while they showed down-regulation at the water temperature of 20°C in comparison to 14°C, which showed fluctuated regulation. The three genes also declined after 24 h post-handling compared to immediately post-handling. Since, oxidative stress prompts under stressful conditions (Chihuailaf et al., 2002) and in general, under situations when high energy demand is not fulfilled (Goff and Horst, 1997). The results of this study came in agreement with that *gpx3, and sod1* genes are upregulated after 2 h post-handling, and returning to their pre-crowding levels after that (Caipang et al., 2008b). Subsequently, developing molecular oxidative stress biomarkers will be useful for early diagnosis of stress which leads to taking further steps to counteract the stress and avoid economic loss in yellow perch, that is why this study was carried out.

When normal cellular processes are adversely affected, a set of proteins belonging to the Hsps are rapidly synthesized, which is called stress proteins as their upregulation is often observed when fish are subjected to stressors including osmotic and heat stress (Iwama et al., 1998). Also, the salinity of the aquatic environment may itself determine whether the heat shock response of an organism is augmented or attenuated upon exposure to stressors (Deane et al., 2002). Here, we showed that salt treatment at 5 ppt for 72 h caused transient Hsp70 down-regulation in 26°C groups in comparison with the control group. Furthermore, continuous salt treatment for 144 h increased Hsp70 expressions; the water temperature 20°C has the minimum effect of salt treatment on Hsp70. Our st (De Wachter et al., 1998)ted by that hepatic HSP-70 levels were significantly elevated in freshwater carp when exposed to pond water supplemented with 1% NaCl, but the brain, gill, and muscle Hsp70 levels were unaltered (De Wachter et al., 1998). In Black sea bream (*Mylio macrocephalus*) hepatic *hsp70* was up regulated at high salinities and down-regulated at isosmotic salinity (Deane et al., 2002). Also, Hsp70 was substantially increased in gills taken from seawater and hypersaline-adapted sea bream (Deane and Woo, 2004) and it was publicized that hyperosmotic exposure of Atlantic salmon gills caused Hsp70 induction within 12 h (Smith et al., 1999).

Energy metabolism and growth in teleost fish are under complex endocrine control that directly or indirectly involves several hormones (Bjornsson et al., 2002). Igf1 widely accepted as being necessary for salinity stress adaptation (Sakamoto and McCormick, 2006; Deane and Woo, 2009). In this study, Igf1 and *igf1* showed that salt treatment caused an alteration in expression through decreasing the stress strength level at 72 h, but continuous salt treatment up to 144 h increased the stress level compared to the control groups. Other studies found that hepatic *igf1* gene abundance was significantly up regulated in isosmotic adapted black sea bream, while hypersaline and hypoosmotic fish showed low expression levels (Deane et al., 2002). Also, expression of hepatic *igf1* gene demonstrated that the highest regulation occurred in isosmotic salinity-adapted and reduced groups in both hypoosmotic and hypersaline-adapted sea bream (Deane and Woo, 2004). Therefore, *Igf1* can use in combined with other stress biomarkers for optimizing the salinity concentration for each fish species. The salt treatment is standard practice in the aquaculture industry, in yellow perch, the farmers always use salt treatment as routine work to avoid infection. So optimizing the salt concentration and the treatment time is critical to prevent more stressful conditions.

Since stress induced by changes in salinity has been related to induced ROS generation, it may seriously affect immune function and lead to oxidative stress, which would impact the fish health status and diseases resistance (Paital and Chainy, 2010; Shin et al., 2010). The present study assessed the effect of salt treatment on oxidative stress markers on protein and gene levels, gpx3, sod1, and gsr and data showed that these markers at 72 h/ST group were upregulated in 26°C, but with a lower expression than 72 h/control groups. However, after 144 h of salt treatment, the opposite was recorded, and the treated group showed a higher up-regulation than the control group. While, at water temperatures of 14 and 20°C, antioxidant genes did not show an increase in the treated group after 144 h compared with 26°C. The expression level of *sod1* and *gpx* were upregulated in black porgy after exposure to hypoosmotic stress (An et al., 2010), and another one found that the expression of *gpx* mRNA in olive flounder increased by exposure to a hypoosmotic environment (Choi et al., 2008). In this study, antioxidant genes were increased at 26°C in the salt treatment group, this is because high temperature and hypoosmotic stresses might have produced plenty of reactive oxygen species in liver, which could have induced SOD to scavenge superoxide radicals and *Gpx* and *Gsr*, to removed H_2_O_2_ (Chae et al., 1999; An et al., 2010; Li et al., 2010). These results suggest that alterations in water salinity could increase the osmotic stress in teleosts. Thereby, maintaining water and ionic homeostasis is of vital importance, as changes in water temperature or salinity impose problems of metabolism alteration, severe ion depletion, alongside alteration of water entry and hemostasis. Furthermore, probiotics show a promise in improving the aquatic animal health and increasing the stress tolerance (Eissa et al., 2010, 2014; Eissa and AbouElgheit, 2011; Mohapatra et al., 2013; Eissa and Abou-ElGheit, 2014). Therefore, future studies are required to investigate this aspect.

The physiological stress response that includes cortisol and catecholamine responses allows fish to maintain their homeostasis, which is necessary for the physiological processes, such as growth, reproduction, survival, and the communication between the central nervous system and immune system (Wendelaar Bonga, 1997; Jentoft et al., 2002; Wilkinson et al., 2006; Prunet et al., 2008; Tort, 2010; Tort and Teles, 2012; Mohapatra et al., 2013; Eissa et al., 2017). While, Oxidative stress response enables fish to mitigate the harmful ROS by promoting the defense system that includes (Gpx, Sod1, Gsr) (Nordberg and Arner, 2001; Chihuailaf et al., 2002; Pandey et al., 2003; Hansen et al., 2006; Lykkesfeldt and Svendsen, 2007; Choi et al., 2008; Li et al., 2010). Several studies reported that existence of the close association between the physiological stress responses and oxidative stress responsive molecules (Miller et al., 2007; Taylor et al., 2016; Birnie-Gauvin et al., 2017). Here, we showed that oxidative stress responses are modulated in response handling at different water temperatures and salt treatment, which is closely similar to our previous study on the physiological stress response in yellow perch (Eissa and Wang, 2013).

Using various genomic and proteomic approaches leads to not only the sighting of new facets in the link between stressors and stress responses but also provides an integrated picture of fish health and welfare. The stress biomarkers have some hitches in the interpretation of results, and searching and understanding alternative tools are crucial for aquaculture, and fish health (Eissa and Wang, 2016). Therefore, we quantified not only the serum and hepatic protein levels of Hsp70, Igf1, Gpx, Sod1, and Gsr but also their mRNA levels in liver tissues of yellow perch were assessed. Consequently, the current study provides potential molecular biomarkers for diagnosing the stress/welfare condition in yellow perch at the earliest onset to reduce disease and increase production. Furthermore, the integration of proteomics, transcriptomics, functional genomics and physiological tools in yellow perch specific stress responses madeveloping y emerge new transcriptomics and proteomics novel ideas linked with the physiology and behavior of this fish species.

The current study has shown that stressors commonly associated with husbandry and environments have effects on the expression of stress related biomarkers in yellow perch. The data findings suggest that the regulation and expression of these biomarkers may be valuable indicators of the physiological state in yellow perch and that they have the potential to improve our understanding of the mechanisms by which yellow perch respond to environmental and husbandry stressors. Salt treatment could minimize stress response caused by handling, but prolonged treatments have an adverse effect on yellow perch and can exaggerate the stress response. Further studies investigating responses to stress over longer periods will be useful in evaluating the time taken for yellow perch to recover from the effects of stress at a cellular and proteomic level. Information on the stress-associated molecules with various stressors in this species could provide relevant new insights into the stress responses that affect fish survival in the aquaculture and recreational fisheries industries.

## Author contributions

Concept and design of the experiments: HW and NE. Conduction of the wet, lab experiments and data analysis: NE. Data collection and handling: NE. Performed research: HY, ZS, AS, and EA. Wrote the manuscript NE and HW. All authors have read and approved the manuscript.

## Conflict of interest statement

The authors declare that the research was conducted in the absence of any commercial or financial relationships that could be construed as a potential conflict of interest.
